# The Delayed Diagnosis of a Submassive Pulmonary Embolism Following Influenza A Infection Treated With Half-Dose Tenecteplase: A Case Report

**DOI:** 10.7759/cureus.67392

**Published:** 2024-08-21

**Authors:** Mohammad Vahid Jorat, Abhirami Shankar, Christine Garabetian, Marie J Geha, Michael Bogseth, Benjamin F Chou

**Affiliations:** 1 Internal Medicine, West Anaheim Medical Center, Anaheim, USA; 2 Emegency Medicine, West Anaheim Medical Center, Anaheim, USA

**Keywords:** submassive pulmonary embolism, tenecteplase, half dose systemic thrombolytic therapy, saddle pulmonary embolism, influenza a infection

## Abstract

The coronavirus 2019 (COVID-19) pandemic has brought renewed attention to thrombotic complications arising from respiratory viral infections, driven by inflammatory responses and activation of the coagulation cascade. While influenza typically resolves on its own, information about its thromboembolic risks remains limited. The persistence of symptoms and the similarity between influenza symptoms and those of pulmonary embolism (PE) often lead to diagnostic delays, which can significantly impact patient outcomes. We discuss a case of a 62-year-old woman with a history of hypertension and hyperlipidemia who presented with dyspnea, syncope, and persistent symptoms following influenza A infection. Despite initial treatment with Tamiflu and subsequent antibiotics for presumed pneumonia, her condition worsened, leading to syncope and admission to the hospital. A diagnostic workup revealed saddle pulmonary emboli with right ventricular strain. She was treated with a half-dose of tenecteplase, resulting in significant clinical improvement and a notable reduction in thrombus burden on follow-up imaging.

The thromboembolic complications of influenza A are not extensively documented compared to COVID-19, yet they can present with severe outcomes. Submassive PE, characterized by hemodynamic stability with signs of right ventricular strain, poses treatment challenges. While full-dose thrombolytics are generally avoided in intermediate-risk PE due to bleeding risks, half-dose thrombolytics such as tenecteplase can offer a safer alternative. Though not FDA-approved specifically for PE, tenecteplase has shown efficacy and was effectively used in this case. Half-dose thrombolytics, including tenecteplase, may be a viable treatment option for certain cases of PE, offering a balance between efficacy and safety. This case highlights the importance of considering PE in patients with persistent respiratory symptoms post-influenza and demonstrates the potential of tenecteplase in managing submassive PE.

## Introduction

Since the emergence of the coronavirus 2019 (COVID-19) pandemic, which highlighted the role of inflammatory and coagulation cascades in viral infections, there has been an increased interest in the thrombotic complications associated with respiratory viral infections. There is currently limited data in the literature on the thromboembolic complications of Influenza, usually a self-limiting viral infection [1]. The similarity in respiratory symptoms between influenza and pulmonary embolism (PE) often leads to delayed diagnosis, which can have dangerous consequences for patients [1,2]. We present a case of a patient with prolonged respiratory symptoms following an influenza A infection, who was eventually diagnosed with submassive pulmonary emboli and treated successfully with a half-dose of tenecteplase.

## Case presentation

A 62-year-old woman with a history of hypertension and hyperlipidemia presented with dyspnea and presyncope. Three weeks before the current admission, she had been diagnosed with influenza A and treated with a full course of Tamiflu as an outpatient. Despite receiving therapy, she continued to experience fever, respiratory difficulty, and cough. She had been admitted to another hospital for community-acquired pneumonia and treated with Levaquin but had remained symptomatic after discharge.

**Figure 1:**
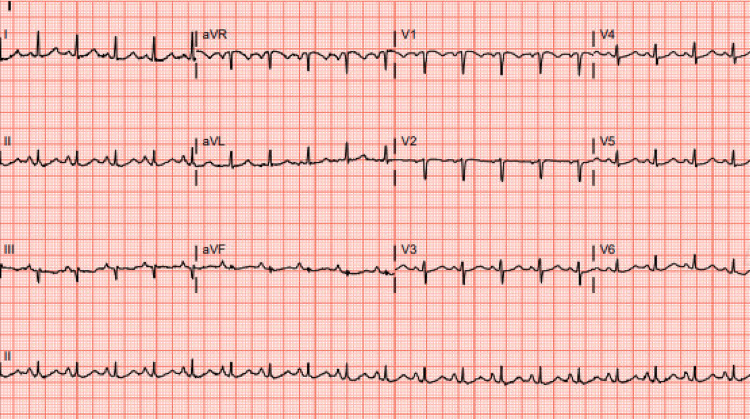
ECG showing sinus tachycardia with S1Q3T3 pattern ECG shows sinus tachycardia with a small S wave in lead I, Q wave, and T wave inversion in lead III, which indicates an S1Q3T3 pattern

Five days later, she experienced syncope while taking a shower and was brought to our hospital. On examination, she was noted to have shortness of breath with a heart rate of 120 bpm, a respiratory rate of 24 breaths per minute, and a blood pressure of 105/54 mmHg. Her oxygen saturation was 99% on a 3L nasal cannula. She had normal breath sounds bilaterally. ECG revealed sinus tachycardia with an S1Q3T3 pattern (Figure 1).

Laboratory tests showed a hypersensitive troponin level at 106 ng/L (reference level: <51 ng/L) and a BNP of 534 pg/L (reference level: <100 pg/L). CT angiography revealed saddle pulmonary emboli with right ventricular (RV) dilatation.

**Figure 2:**
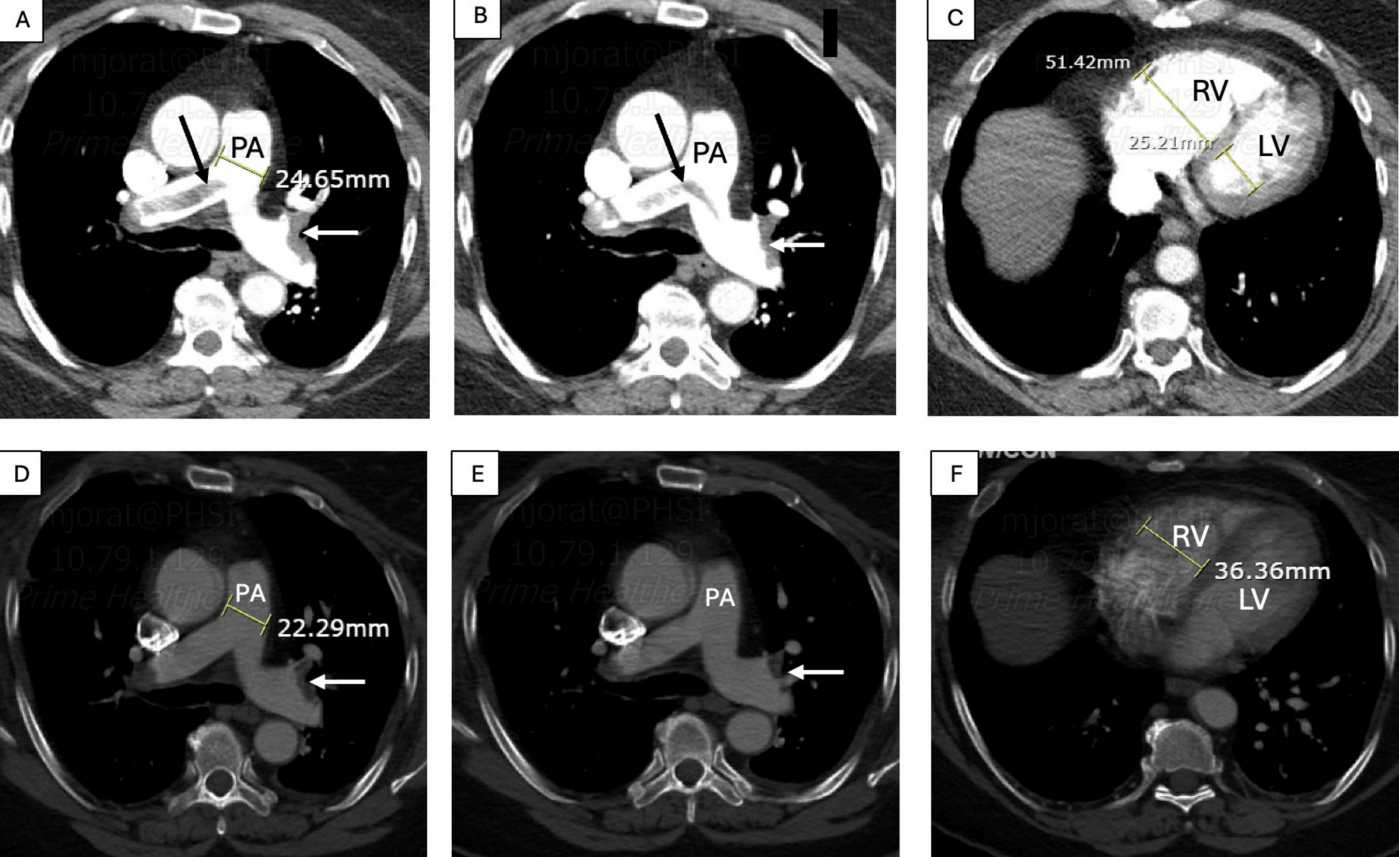
Chest CTA before and after receiving tenecteplase A, B, and C: CTA before thrombolysis: saddle pulmonary emboli with extension to bilateral pulmonary arteries (black arrow) and subsegmental branches with pulmonary artery (PA) and right ventricle (RV) dilatation; PA diameter: 24.65 mm, RV diameter: 51.42 mm. D, E, and F: CTA three days after thrombolysis: significant reduction in thrombus burden with residual thrombus in distal pulmonary arteries and lower lobe branches (white arrow) and a significant decrease in PA and RV dimensions; PA diameter: 22.29 mm, RV diameter 36.36 mm, which indicate successful thrombolysis CTA: computed tomography angiography

The patient was administered a half dose of tenecteplase. Her symptoms improved, and tachycardia resolved a few hours after thrombolysis. A follow-up CT angiography performed three days later at the patient's request showed a significant reduction in bilateral thrombus burden, with residual bilateral thrombi in the distal main and lower lobe branches (Figure 2). She had no other predisposing factors for thrombosis and experienced no further complications. She was discharged on oral anticoagulation after five days.

## Discussion

There is limited data in the literature regarding the thromboembolic complications of influenza A, which are less common compared to COVID-19 infection [1]. As per available data, deep vein thrombosis (DVT) and PE are the most common complications [1]. Current guidelines recommend systemic thrombolysis, catheter-directed thrombus removal with or without thrombolysis, or surgical embolectomy for hemodynamically unstable patients [2]. Submassive pulmonary emboli are characterized by hemodynamically stable PE accompanied by pulmonary hypertension on echocardiography, RV cavity expansion, interventricular septal deviation, or a right-to-left ventricular ratio equal to or greater than 0.9 on CT angiography, with or without elevated troponin and BNP levels [2].

Various studies, including a meta-analysis by Zhang et al., do not recommend full-dose thrombolytics for patients with intermediate-risk PE, including submassive embolism, due to the higher bleeding risk outweighing its benefits [3]. Half-dose alteplase can prevent death or hemodynamic decompensation in the first seven-day and 30-day periods compared with low-molecular-weight heparin (LMWH) treatment, without increasing the risk of bleeding [3-4]. There are no studies in the literature comparing the efficacy and long-term outcome of tenecteplase with other thrombolytics. Although the FDA has not approved this drug for the treatment of PE, some studies have shown its efficacy [5]. Tenecteplase, due to its longer half-life, can be administered as a single bolus dose more easily than other thrombolytics [5]. It was used effectively in our case without any complications.

## Conclusions

PE is a potential complication of influenza A that should be considered in patients with persistent respiratory difficulty and oxygen desaturation after initial treatment. A high index of suspicion and the use of appropriate diagnostic tools can prevent delayed diagnosis and potential complications. Half-dose thrombolytics, including tenecteplase, can be considered in the treatment of selective cases of submassive pulmonary emboli, as well as massive PE without contraindications.
